# Advances in the Study of the Structures and Bioactivities of Metabolites Isolated from Mangrove-Derived Fungi in the South China Sea

**DOI:** 10.3390/md11103601

**Published:** 2013-09-30

**Authors:** Xin Wang, Zhi-Gang Mao, Bing-Bing Song, Chun-Hua Chen, Wei-Wei Xiao, Bin Hu, Ji-Wen Wang, Xiao-Bing Jiang, Yong-Hong Zhu, Hai-Jun Wang

**Affiliations:** 1 Department of Neurosurgery and Pituitary Tumour Center, the First Affiliated Hospital of Sun Yat-sen University, No. 74, Zhongshan Road 2, Guangzhou 510080, China; E-Mails: wangx357@mail2.sysu.edu.cn (X.W.); mmh222111@yahoo.com.cn (Z.-G.M.); warm0809@qq.com (B.H.); 104956978@qq.com (J.-W.W.); 81939338@qq.com (X.-B.J.); 2 Department of Histology and Embryology, Medical school of Sun Yat-sen University, No. 74, Zhongshan Road 2, Guangzhou 510080, China; E-Mails: hd_sbb@163.com (B.-B.S.); 1149755456@qq.com (C.-H.C.); xiaoweiweigz@163.com (W.-W.X.); 3 Key Laboratory of Functional Molecules from Marine Microorganisms, Department of Education of Guangdong Province, Sun Yat-sen University, No. 74, Zhongshan Road 2, Guangzhou 510080, China

**Keywords:** marine metabolites, fungus, mangrove, biological activity, south China sea

## Abstract

Many metabolites with novel structures and biological activities have been isolated from the mangrove fungi in the South China Sea, such as anthracenediones, xyloketals, sesquiterpenoids, chromones, lactones, coumarins and isocoumarin derivatives, xanthones, and peroxides. Some compounds have anticancer, antibacterial, antifungal and antiviral properties, but the biosynthesis of these compounds is still limited. This review summarizes the advances in the study of secondary metabolites from the mangrove-derived fungi in the South China Sea, and their biological activities reported between 2008 and mid-2013.

## 1. Introduction

The oceans, which cover more than 70% of the earth’s surface, are not only rich in minerals but also have various marine organisms. The international census of marine life (CoML) has claimed that the marine microorganisms amount to 5–10 million, which is far more than the original estimate of 200,000, and far more than the sum of plant and animal species across the world. However, to date, approximately 5000 kinds of marine microorganisms have been officially named and described. The recent advances in natural product chemistry, underwater exploration, bioassays, and genome mining have stimulated interest in the search for new products from this unexplored wealthy habitat [1]. Nowadays, the marine world is a major source for discovery drugs from natural products [2,3]. Such as Cod-liver oil is rich in vitamins A and D, and it probably was the first marine natural products widely commercialized. Arabinofuranosyl-adenine (Ara-A) was isolated from the gorgonian Eunicella cavolini in 1984, it potent used for antitumor and antiviral therapy in the clinic [4,5]. In addition, cephalosporins, digenicacid, active absorbable calcium, bataan, didemnin B, and Ara-C, have gone into clinical use. The biological diversity of the marine ecosystem has provided many promising compounds. In addition, the marine compounds have potent biological activities such as analgesic, antiallergic, antiviral, anticancer, anti-inflammatory, and immunomodulatory activities [6,7].

Marine-derived fungi are a rich source of promising natural products that have biological activities [8]. The secondary metabolites are a diverse group of organic compounds but most of them do not appear to participate directly in the normal growth, development, or reproduction of an organism [9,10]. With an increase in the research on marine microorganisms, an increasing number of new, bioactive, and structurally unique metabolites are being found from marine fungi. In addition, various studies have shown that mangrove-derived fungi yield many novel or bioactive secondary metabolites that are indispensable for drug development [11,12]. In this review, we have summarized the sources and structures of 110 compounds that have been extracted from mangrove-derived fungi from the South China Sea and focused on their bioactivity reported between 2008 and mid-2013.

## 2. Metabolites Derived from the Mangrove Fungi in the South China Sea

Many bioactive metabolites, including anthracenediones, xyloketals, sesquiterpenoids, chromones, lactones, coumarin and isocoumarin derivatives, xanthones, and peroxides, *etc.* have been isolated from various mangrove-derived fungi in the South China Sea. The sources, marine products, biological activities, and articles related to these products are listed as shown in Table 1.

**Table 1:** Metabolites from diverse mangrove fungi.

Source	Compound	Activity	Ref.
*Halorosellinia* sp. (No. 1403)	SZ-685C (**1**)	Cytotoxic	[13]
	Bostrycin (**16**)	Cytotoxic	[14]
*Halorosellinia* sp. (No. 1403) and *Guignardia* sp. (No. 4382)	Compounds (**2**–**15**)	Cytotoxic (**6**)	[15]
*Nigrospora* sp.	4-Deoxybostrycin (**18**)	Anti-mycobacteria	[16]
	Nigrosporin (**19**)	Anti-mycobacteria	
*Alternaria* sp. (ZJ9-6B)	Alterporriol K (**20**), L (**21**) and M (**22**)	Cytotoxic (**20**,**21**)	[17]
*Paecilomyces* sp.	Secalonic acid A (**23**)	Cytotoxic	[18]
*Paecilomyces* sp. (tree 1–7) and endophytic fungus No. ZSU44	Secalonic acid D (**24**)	Cytotoxic	[19,20,21]
*Xylaria* sp. (No. 2508)	Xyloketal B (**25**)	Protects Human umbilical vein endothelial cells from oxidized LDL-induced oxidative injury	[22]
	Xyloketal J (**26**)		[23]
	Xyloester A (**27**)		
*Xylaria* sp. BL321	Eremophilane sesquiterpenes (**33**–**35**)		[24]
	07H239-A (**36**)	Effect on α-glucosidase	
*Aspergillus* sp.	(+)-methyl sydowate (**28**)	Antibacterial	[25]
	7-deoxy-7,8-didehydrosydonic acid (**29**)		
	7-deoxy-7,14-didehydrosydonic acid (**30**)		
	(+)-sydonic acid (**31**)	Antibacterial	
	(+)-sydowic acid (**32**)	Antibacterial	
*Aspergillus* sp. (16-5c)	Asperterpenoid A (**37**)	Anti-Mycobacterium tuberculosis	[26]
*Aspergillus terreus* Gwq-48	Isoaspulvinone E (**58**), pulvic acid (**59**) and aspulvinone E (**60**)	Anti-influenza A H1N1 virus	[27]
*Diaporthe* sp.	Diaporols A–I (**38**–**46**)		[28]
*Pestalotiopsis* sp.	Pestalotiopsones A–F (**47**–**52**)	Cytotoxic (52)	[29]
	Cytosporones J–N (**68**–**72**)		[30]
	Dothiorelone B (**73**)		
	Pestalasins A–E (**74**–**78**)		
	3-hydroxymethyl-6,8-dimethoxycoumarin (**56**)		
	7-hydroxy-2-(2-hydroxypropyl)-5-methylchromone (**53**)		
*Phomopsis* sp. (ZZF08)	Phomopsin A (**61**)	Cytotoxic	[31]
	Cytochalasin H (**62**)	Cytotoxic	
	Glucosylceramide (**63**)		
*Phomopsis* sp. (ZSU-H76)	Phomopsin A (**61**), B (**64**), C (**65**)		[32]
	Cytosporone B (**66**) and C (**67**)	Antifungal	
*Phomopsis* sp. (No. SK7RN3G1)	2,6-dihydroxy-3-methyl-9-oxoxanthene-8-carboxylic acid methyl ester (**88**)		[33]
*Penicillium* sp. (091402)	(3 R*, 4S*)-6,8-dihydroxy-3,4,7-trimethylisocoumarin (**79**)	Cytotoxic	[34]
	(3 *R*, 4*S*)-6,8- dihydroxy-3,4,5-trimethylisocoumarin (**80**)		
	(3 *R*, 4*S*)-6,8- dihydroxy-3,4,5,7-tetramethylisocoumarin (**81**)		
	( *S*)-3-(3′,5′-dhydroxy-2′,4′-methlphenyl)butan-2-one (**82**)		
	Phenol A (**83**)	Cytotoxic	
*Penicillium* sp. (ZZF 32#)	Dimethyl 8-methoxy-9-oxo-9H-xanthene-1, 6-dicarboxylate (**86**)		[35]
	8-(methoxycarbonyl)-1-hydroxy-9-oxo-9 *H*-xanthene-3-carboxylic acid (**87**)	Antifungal	
*Talaromyces flavus*	Talaperoxides A–D (**89**–**92**)	Cytotoxic	[36]
	Steperoxide B (**93**, or merulin A)	Cytotoxic	
*Talaromyces* sp. (SBE-14)	Tenelate A (**108**) and B (**109**)		[37]
	Tenellic acid C (**110**)		
*Sporothrix* sp. (#4335)	Sporothrins A, B, and C (**105**–**107**)	Cytotoxic	[38]
Unidentified fungus (No. B77)	Anhydrofusarubin (**17**)	Anti-Gram-positive bacteria, Cytotoxic	[39]
Unidentified fungus (No. GX4-1B)	1,10-dihydroxy-8-methyl-dibenz[*b*,*e*]oxepin-6,11-dione (**54**)		[40]
	6-hydroxy-4-hydroxymethyl-8-methoxy-3-methylisocoumarin (**55**)		
	3-hydroxymethyl-6,8-dimethoxycoumarin (**56**)		
	1,10-dihydroxy-dibenz[*b* ,*e*]oxepin-6,11-dione (**57**)		
Unidentified fungus (No. ZH19)	1-hydroxy-4,7-dimethoxy-6-(3-oxobutyl)-9 *H*-xanthen-9-one (**84**)	Cytotoxic	[41]
	1,7-dihydroxy-2-methoxy-3-(3-methylbut-2-enyl)-9 *H*-xanthen-9-one (**85**)	Cytotoxic	

### 2.1. Anthracenediones

**Figure 1:**
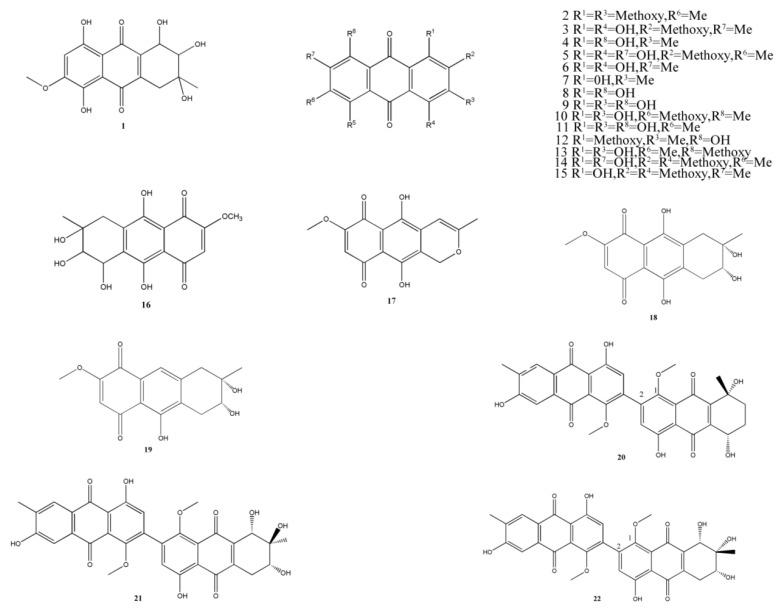
Chemical structures of metabolites anthracenediones.

Anthracenedione is one of the important sources of marine secondary metabolites. They have a great potential of biomedical applications because their novel structures and bioactivities (Figure 1).

A novel marine anthraquinone derivative, SZ-685C (**1**), was isolated from *Halorosellinia* sp. (No. 1403), a mangrove-derived fungus in the South China Sea. A previous study showed that SZ-685C inhibits the growth of six tumor cell lines, including human glioma, hepatoma, prostate cancer, and breast cancer (half-maximal inhibitory concentration [IC_50_] = 3.0–9.6 × 10^3^ μM), and *in vivo* experiments showed that SZ-685C also inhibits the tumor growth in nude mice by inducing apoptosis via the Akt/FOXO pathway [13]. Subsequently, Zhu *et al.* found that compound **1** causes apoptosis in adriamycin-resistant human breast cancer cells both *in vitro* and *vivo*, and it exerts these antitumor effects through multiple mechanisms mainly involving the suppression of Akt signaling [42]. Recently, Chen *et al.* [43] reported that SZ-685C significantly inhibited the proliferation of MMQ pituitary adenoma cells and induced apoptosis by downregulation of miR-200c. In addition, this compound showed potent anticancer activity in radiosensitive and radioresistant nasopharyngeal carcinoma cells, and the miR-205-PTEN-Akt pathway is the mechanism underlying the anticancer activity [44]. Zhang *et al.* isolated 14 anthracenedione derivatives (**2**–**15**) from the mangrove fungi *Guignardia* sp. (No. 4382) and *Halorosellinia* sp. (No. 1403). Of these compounds, compound **6** was effective against KBv200 cells and KB cells (IC_50_ = 3.21 and 3.17 μM, respectively) and induced apoptosis through a mitochondrial pathway [15].

Xu *et al.* [14] showed that an anthracenedione with phytotoxic properties, bostrycin (**16**), was collected from the same strain *Halorosellinia* sp. (No. 1403). They showed that this compound was cytotoxic against yeast cells, and it might induce apoptosis through a mitochondria-mediated apoptotic pathway. Another novel anthraquinone, anhydrofusarubin (**17**) isolated from the endophytic fungus No. B77, showed an inhibitory effect on the gram-positive bacterium *Staphylococcus aureus* (ATCC27154; minimum inhibitory concentration [MIC] = 43.4 μM) [45] and showed a significant inhibition of the growth of HEp2 and HepG2 cells (IC_50_ = 8.67 and 3.47 μM, respectively) [39].

A natural anthraquinone, 4-deoxybostrycin (**18**), and its deoxy derivative, nigrosporin (**19**), were obtained from *Nigrospora* sp. A primary bioassay showed that both compounds showed inhibitory effects against mycobacteria, and compound **18** showed significant inhibition of some clinical multidrug-resistant (MDR) *Mycobacterium Tuberculosis* strains (MIC < 15.7 μM) [16]. Huang *et al.* isolated alterporriol K (**20**), L (**21**), and M (**22**), three new bianthraquinone derivatives, from the endophytic mangrove fungus *Alternaria* sp. ZJ9-6B. Of these three derivatives, compounds **20** and **21** were moderately active against MDA-MB-435 and MCF-7 human breast cancer cell lines (IC_50_ = 13.1–29.1 μM) [17].

### 2.2. Secalonic Acid Family

**Figure 2:**
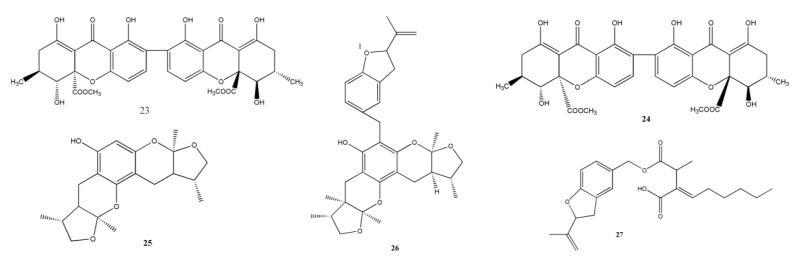
Chemical structures of metabolites secalonic acid family and xyloketals.

Secalonic acid A (SAA) (**23**) was isolated from the marine fungi *Paecilomyces* sp. and has been reported to have strong antitumor activities (Figure 2). Zhai *et al.* [18] found that pretreatment with SAA has neuroprotective effects in cultured rat cortical neurons, which was associated with the suppression of c-Jun N-terminal kinase (JNK), calcium influx, p38 mitogen-activated protein kinase (MAPK), and the activation of caspase-3. Moreover, compound **23** may rescue dopaminergic neurons from 1-methyl-4-phenylpyridinium (MPP+)-induced cell death through the mitochondrial apoptotic pathway, and this protective effect of **23** was observed in mice with Parkinson’s disease [46].

Another promising metabolite, secalonic acid D (SAD) (**24**), was initially isolated from *Penicillium oxalicum* by Steyn PS in 1969 and was investigated as a teratogenic fungal metabolite with strong toxicity [47]. In the following decades, studies on SAD mainly focused on its toxicity in mice. Several studies showed that SAD could cause cleft palate through the inhibition of G1/S-phase-specific CDK2 activity [48]. SAD inhibited the proliferation of human embryonic palatal mesenchymal cells by suppressing cell cycle progression and altering the expression of cell cycle regulatory proteins, p21 and cyclin E [49].

Recent studies have shown that SAD has strong anticancer activities. Hong [50] showed that SAD might act as a novel DNA topoisomerase I inhibitor (MIC = 0.4 µM) and be a potential anticancer drug; SAD was separated from the fermentation broth of marine sediments-derived fungi *Gliocladium* sp. T31 obtained from the South Pole.

Some studies have shown that SAD was extracted from the secondary metabolites of the mangrove-derived fungi No. ZSU44. Zhang *et al.* [19] found that SAD showed significant cytotoxic activities and induced apoptosis in K562 and HL60 myeloid leukemia cell lines (IC_50_ = 0.43 and 0.38 μM, respectively), and it exerted this effect by blocking the G1 phase of the cell cycle through the GSK-3β/β-catenin/c-Myc pathway. Recently, Hu *et al.* [20] suggested that SAD was active against MDR cells and reduced the percentage of side population cells in lung cancer through downregulation of the expression levels of ABCG2. Liao *et al.* [21] reported that SAD was isolated from *Paecilomyces* sp. (tree 1–7); SAD inhibited the proliferation of murine pituitary adenoma GH3 cells and induced apoptosis in a dose-dependent manner. SAD exerted its cytotoxic effect mainly through cell cycle arrest by activating caspase. In addition, SAD inhibited the expression of growth hormone in GH3 cells.

### 2.3. Xyloketals

A novel bioactive marine compound, xyloketal B (**25**), has been isolated from the mangrove fungus *Xylaria* sp. (no. 2508). Chen *et al.* showed for the first time that xyloketal B protected human umbilical vein endothelial cells from oxidized low-density lipoprotein-induced oxidative injury by suppressing NADPH oxidase-derived generation of reactive oxygen species, recovering the expression levels of Bcl-2, and increasing nitric oxide (NO) production [22]. Subsequently, Zhao *et al.* [51] found that compound **25** could protect rat pheochromocytoma PC12 cells against oxygen glucose deprivation (OGD)-induced cell damage.

In the same mangrove fungus, Xu *et al.* [23] found two metabolites, xyloketal J (**26**) and xyloester A (**27**). Compound **26** showed results similar to those observed in compound **25**; however, in their primary bioactivity test, the two metabolites were inactive against bacteria.

### 2.4. Sesquiterpenoids

**Figure 3:**
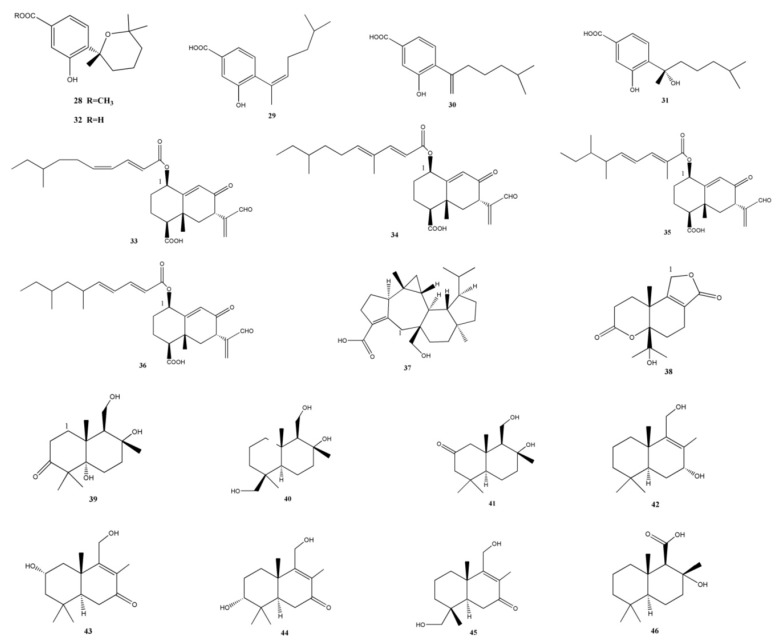
Chemical structures of metabolites sesquiterpenoids.

Wei *et al.* obtained three new phenolic bisabolane-type sesquiterpenoids along with two known fungi-derived metabolites from the marine-derived fungus *Aspergillus* sp. (Figure 3). They were (+)-methyl sydowate (**28**), 7-deoxy-7,8-didehydrosydonic acid (**29**), 7-deoxy-7,14-didehydrosydonic acid (**30**), (+)-sydonic acid (**31**), and (+)-sydowic acid (**32**). Out of which, compound **28**, **30**, and **31** were weakly active against *S. aureus*, however, they displayed inactive effects against methicillin-resistant *S. aureus* [25]. Song *et al.* reported three new eremophilane sesquiterpenes (**33**–**35**) along with a known analogue 07H239-A (**36**) obtained from the marine-derived fungus *Xylaria* sp. BL321. Out of these, compound **36** activated α-glucosidase at a concentration of 0.15 μM, and compound **36** exhibited an inhibitory effect against α-glucosidase at increased concentrations (IC_50_ = 6.54 μM) [24].

A novel sesterterpenoid, asperterpenoid A (**37**), isolated from a mangrove endophytic fungus *Aspergillus* sp. 16-5c, efficiently inhibited *M. tuberculosis* protein tyrosine phosphatase B (IC_50_ = 2.2 μM) [26]. Another new sesquiterpenoid, diaporol A (**38**), and eight other novel drimane sesquiterpenoids, diaporols B–I (**39**−**46**), were obtained from a culture of the marine-derived endophytic fungus *Diaporthe* sp., but a primary bioassay indicated that they were not cytotoxic [28].

### 2.5. Chromones

**Figure 4:**
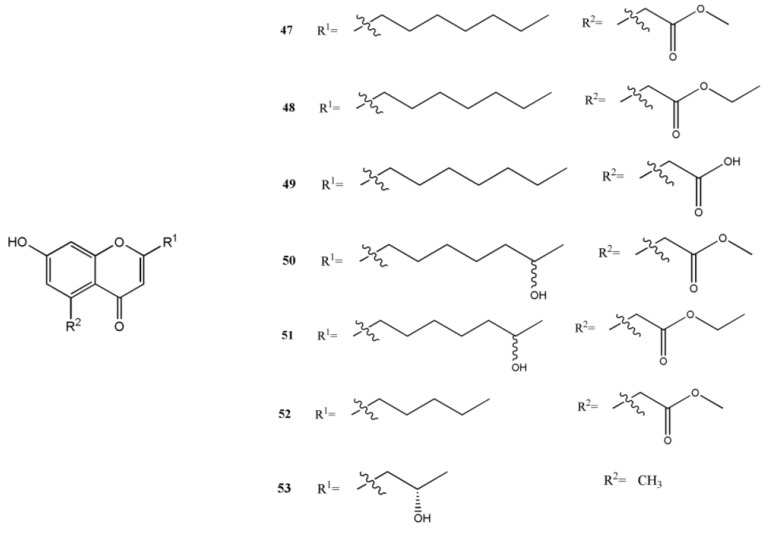
Chemical structures of metabolites chromones.

Pestalotiopsones A–F (**47**–**52**), the new chromones have been reported by Xu *et al.*, together with the known derivative 7-hydroxy-2-(2-hydroxypropyl)-5-methylchromone (**53**) obtained from the mangrove-derived endophyte *Pestalotiopsis* sp. isolated from *Rhizophora mucronata* leaves (Figure 4). A preliminary biological activity test showed that compound **52** was moderately cytotoxic against the mouse L5178Y lymphoma cell line, while the other six metabolites were inactive [29].

### 2.6. Lactones

**Figure 5:**
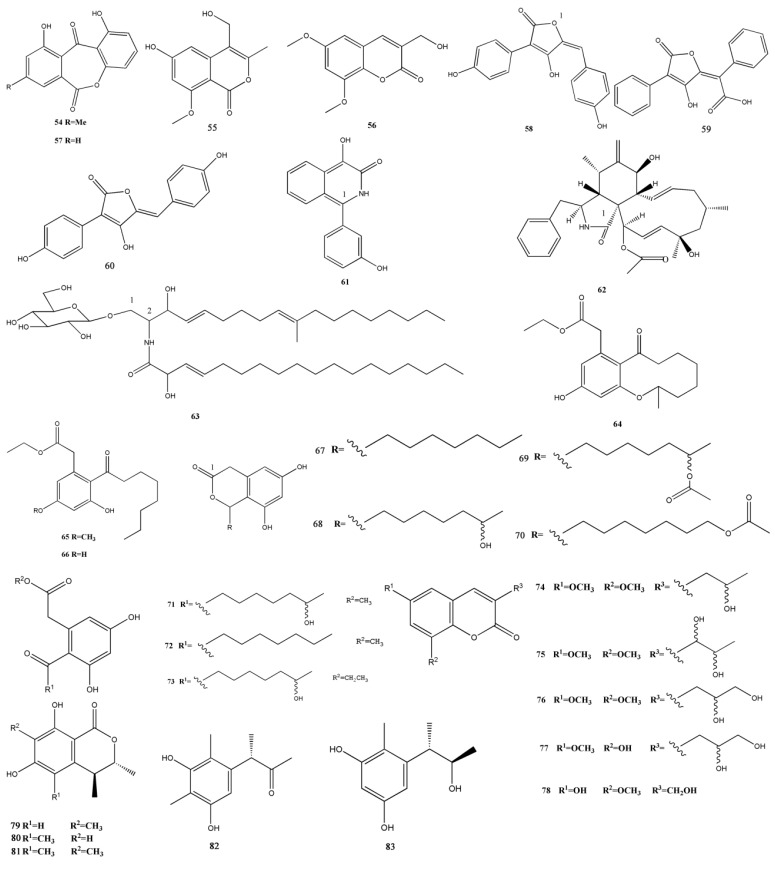
Chemical structures of metabolites lactones, coumarins, and isocoumarin derivatives.

Lactones continue to be a great source for new bioactive natural products (Figure 5). Two new aromatic lactones, 1,10-dihydroxy-8-methyl-dibenz[*b*,*e*]oxepin-6,11-dione (**54**) and 6-hydroxy-4-hydroxymethyl-8-methoxy-3-methylisocoumarin (**55**), along with two known compounds, 3-hydroxymethyl-6,8-dimethoxycoumarin (**56**) and 1,10-dihydroxy-dibenz[*b*,*e*]oxepin-6,11-dione (**57**), were collected from an unidentified endophytic fungus No. GX4-1B, which was obtained from *Bruguiera gymnoihiza* (L.), but their bioactivities have not been examined in this research [40].

Recently, Gao *et al.* [27] isolated a new butenolide isoaspulvinone E (**58**) and two known butenolides pulvic acid (**59**) and aspulvinone E (**60**) from *Aspergillus terreus* Gwq-48. All the three compounds were moderately against influenza A H1N1 virus (IC_50_ = 101.3, 94.5, and 192.2 μM, respectively).

Tao *et al.* [31] isolated a novel marine product named phomopsin A (**61**) along with two known compounds cytochalasin H (**62**) and glucosylceramide (**63**) from the mangrove endophytic fungus *Phomopsis* sp. (ZZF08). Bioactivity assay of these compounds showed that compound **61** was moderately active against KBv200 cells and KB cells (IC_50_ = 66.4 μM and 110.7 μM, respectively) and compound **62** showed significant cytotoxic activity against KBv200 cells and KB cells (IC_50_ < 2.5 μM). Huang *et al.* [32] found phomopsin B (**64**) and C (**65**) from the endophytic fungus, *Phomopsis* sp. ZSU-H76, along with two known compounds cytosporone B (**66**) and C (**67**) from the stem of a Chinese mangrove plant *Excoecaria agallocha*. Compounds **66** and **67** inhibited two fungi *Fusarium oxysporum* and *Candida albicans* (MIC = 115.1–198.8 μM). Cytosporones J–N (**68**–**72**) and two known compounds, dothiorelone B (**73**) and cytosporone C (**67**) were obtained from the mangrove fungi *Pestalotiopsis* sp. [30]. Compound **68**–**72** were inactive against cancer cells.

### 2.7. Coumarins and Isocoumarin Derivatives

Mangrove fungi isolated from the South China Sea also yielded a variety of coumarins and isocoumarin derivatives with novel structures.

Five novel coumarins, pestalasins A–E (**74**–**78**), and a known compound 3-hydroxymethyl-6,8-dimethoxycoumarin (**56**) were isolated from the Chinese mangrove *Rhizophora mucronata*-derived *Pestalotiopsis* sp. [30]. These compounds were isolated from a mangrove fungus for the first time by Xu *et al.* A new compound (3R*, 4S*)-6,8-dihydroxy-3,4,7-trimethylisocoumarin (**79**) was obtained from the mangrove endophytic *Penicillium* sp. 091402 from the plant *Bruguiera sexangula* together with four known derivatives (3*R*, 4*S*)-6,8-dihydroxy-3,4,5-trimethylisocoumarin (**80**) and (3*R*, 4*S*)-6,8-dihydroxy-3,4,5,7-tetramethylisocoumarin (**81**), (*S*)-3-(3′,5′-dihydroxy-2′,4′-methlphenyl) butan-2-one (**82**), and phenol A (**83**), the structures of which were consistent with that of the decomposition product of citrinin. Of these compounds, compound **80** was cytotoxic against cancer cell line K562 (IC_50_ = 84.7 μM), and compound **83** showed weak cytotoxicity against the cancer cell line SGC-7901(IC_50_ = 195.7 μM) [34].

### 2.8. Xanthones and Peroxides

**Figure 6:**
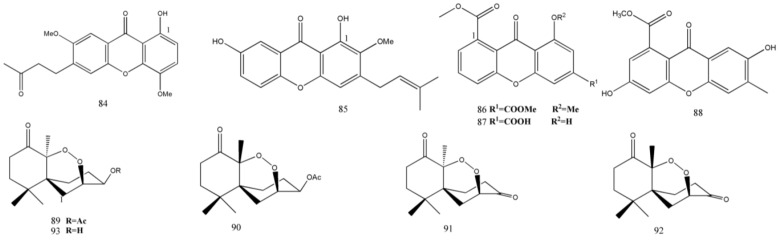
Chemical structures of metabolites xanthones and peroxides.

Xanthones and peroxides play an important role in the source of promising drugs (Figure 6). They produce several structurally diverse, bioactive metabolites.

Huang *et al.* [41] isolated two novel xanthone derivatives, 1-hydroxy-4,7-dimethoxy-6-(3-oxobutyl)-9*H*-xanthen-9-one (**84**) and 1,7-dihydroxy-2-methoxy-3-(3-methylbut-2-enyl)-9*H*-xanthen-9-one (**85**), from a mangrove endophytic fungus (No. ZH19). Compounds (**84**) and (**85**) inhibited KB cells (IC_50_ = 3.5 × 10^4^ and 2.0 × 10^4^ μM, respectively) and KB(V)200 cells (IC_50_ = 4.1 × 10^4^ and 3.0 × 10^4^ μM, respectively). Dimethyl 8-methoxy-9-oxo-9*H*-xanthene-1, 6-dicarboxylate (**86**), and 8-(methoxycarbonyl)-1-hydroxy-9-oxo-9*H*-xanthene-3-carboxylic acid (**87**) were other novel xanthones isolated from the culture broth of the mangrove fungus *Penicillium* sp. (ZZF 32#) from the South China Sea. Among these, compound **87** was active against *Fusarium oxysporum* f. sp. *cubense* with moderate antifungal activity (MIC = 39.8 μM) [35].

Recently, a novel xanthone, 2,6-dihydroxy-3-methyl-9-oxoxanthene-8-carboxylic acid methyl ester (**88**), was isolated from the marine fungus *Phomopsis* sp. (No. SK7RN3G1) by Yang *et al.* Primary bioassays showed that compound **88** was cytotoxic against HepG2 and HEp-2 cells (IC_50_ = 30 and 26.7 μM, respectively) [33].

Talaperoxides A–D (**89**–**92**), four new norsesquiterpene peroxides, and steperoxide B (**93**), a known analogue, were isolated from the mangrove-derived fungus *Talaromyces flavus*. All the four compounds, in particular compounds **90** and **92**, were cytotoxic against several human tumor cell lines HeLa, HepG2, MCF-7, PC-3, and MDA-MB-435 (IC_50_ = 2.8–9.4 μM) [36].

### 2.9. Other

**Figure 7:**
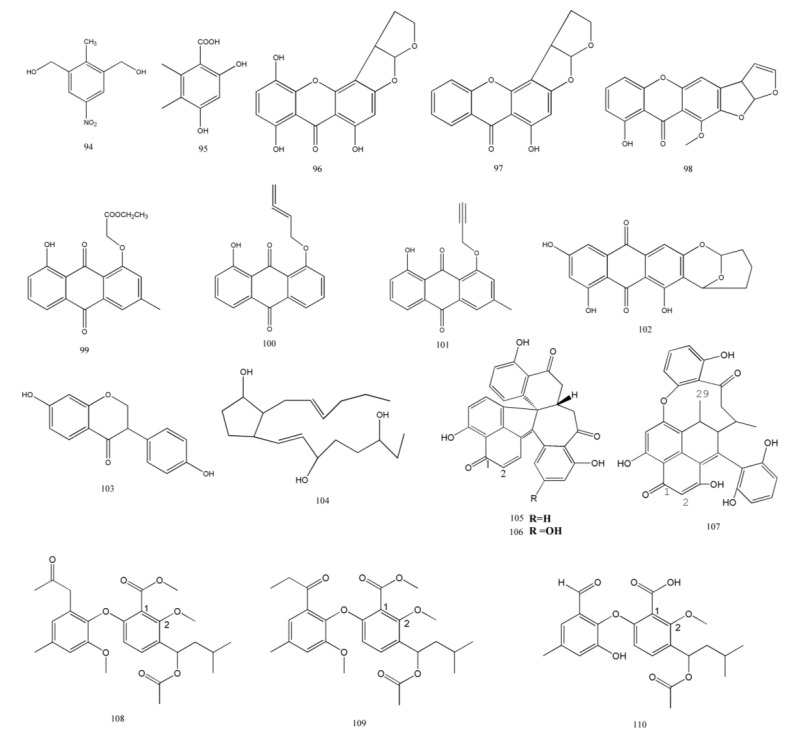
Chemical structures of other metabolites.

There are several other metabolites with biological activities (Figure 7). Tao *et al.* examined 87 natural products isolated from the mangrove fungus in the South China Sea [52]. From these products, 11 (**94**–**104)** showed potent cytotoxicity in KBv200, A549, KB, MCF-7/adr, and MCF-7 cells (IC_50_ < 50 μM). Compared to normal liver cells, the compounds **94**, **102**, **103**, and **104** were more sensitive against cancer cells in this research (IC50 were at least 1.35 fold more potent against cancer cells). In addition, while compound **95** and **99** showed complete inhibition of the growth of LO2 cells, the other compounds were inactive.

Sporothrins A, B, and C (**105**–**107**), three bioactive metabolites, were collected from the mangrove fungus *Sporothrix* sp. (#4335). Compound **105** and **106** showed moderate cytotoxic activity against HepG2 cell lines (IC_50_ = 108.2 and 41.8 μM, respectively). Moreover, compound **105** significantly inhibited the activity of acetylcholine esterase (AChE) *in vitro* (IC_50_ = 1.05 μM) [38]. Liu *et al.* [37] identified two novel metabolites and known compounds named tenelate A (**108**), B (**109**), and tenellic acid C (**110**); these compounds were separated from the marine-derived fungus *Talaromyces* sp. (SBE-14). The bioactivities of these compounds have not been examined in this study.

## 3. Conclusions

The oceans are the largest underexploited wealthy resource of potential drugs. Mangrove fungi are increasingly being explored in the studies on novel and bioactive molecules from marine sources. Several new bioactive compounds of potential therapeutic have been isolated and identified from mangrove fungi during the last 5 years. Some of them have a high toxicity toward cancer cells; however, they are also cytotoxic to normal cells. These compounds require structural modifications to decrease the toxicity and increase the anticancer activity. In addition, the bioactivity of the compounds was mainly examined *in vitro*; thus, further *in vivo* and preclinical studies are required to determine the bioactive compounds with potential therapeutic applications. The side effects of these metabolites should be examined in future studies.

Further, for the industrial large-scale production of these metabolites, there is an important hurdle that mass culture of the fungi and compound purification. Because the biosynthesis of these compounds is limited, large amounts of active materials cannot be obtained from mangrove fungi. In recent research, this may mean increased production through changing the function of the genes and enzymes, seed culture methods, and larger field of the metabolic engineering of culturable microorganisms. With the technical advancements in isolation and cultivation of marine microorganisms, we infer that marine natural products will lead to a new surge of drugs, and fungi and other marine microorganisms will be promising sources for novel therapeutic agents.
